# Supplementary material for: Effects of neuromuscular gait modification strategies on indicators of knee joint load in people with medial knee osteoarthritis: A systematic review and meta-analysis
Source: PLoS One. 2022 Sep 21;17(9):e0274874. doi: 10.1371/journal.pone.0274874 (PMC9491578; doi:10.1371/journal.pone.0274874)
Supplement: S1 Appendix — (DOCX) [file pone.0274874.s001.docx]

**S1 Appendix:** Search strategy (in MEDLINE database)

| **#** | **Searches** |
| --- | --- |
| 1 | exp Osteoarthritis/ |
| 2 | exp Osteoarthritis, Knee/ |
| 3 | (osteoarthr* or osteo arthr* or osteo- arthr*).mp. |
| 4 | (degenerat* adj2 joint*).mp. |
| 5 | 1 or 2 or 3 or 4 |
| 6 | exp knee/ |
| 7 | *knee/ |
| 8 | genu.mp. |
| 9 | (tibiofemoral or tibio- femoral or tibio femoral).mp. |
| 10 | (medial adj4 knee*).mp. |
| 11 | (knee* adj4 joint*).mp. |
| 12 | 6 or 7 or 8 or 9 or 10 or 11 |
| 13 | 5 and 12 |
| 14 | exp Gait/ |
| 15 | exp Gait Analysis/ |
| 16 | (gait* or walk* or ambulat* or locomot*).mp. |
| 17 | (gait adj (disorder* or deviat*)).mp. |
| 18 | 14 or 15 or 16 or 17 |
| 19 | 13 and 18 |
| 20 | exp Rehabilitation/ |
| 21 | Rehabilitation Centers/ or Rehabilitation Research/ or Hospitals, Rehabilitation/ or Rehabilitation Nursing/ or "Physical and Rehabilitation Medicine"/ |
| 22 | (train* or retrain* or re- train* or modif* or educat* or reeducat* or re- educat* or intervent* or strateg* or pattern* or rehabilitat*).mp. |
| 23 | 20 or 21 or 22 |
| 24 | 19 and 23 |
| 25 | exp Biomechanical Phenomena/ |
| 26 | (biomechanic* or bio- mechanic* bio mechanic*).mp. |
| 27 | kinematic*.mp. |
| 28 | knee* adduct* moment*.mp. |
| 29 | KAM.mp. |
| 30 | varus thrust*.mp. |
| 31 | (knee joint adj load*).mp. |
| 32 | (joint load* or load* or moment* or force*).mp. |
| 33 | 25 or 26 or 27 or 28 or 29 or 30 or 31 or 32 |
| 34 | 24 and 33 |
| 35 | exp Adult/ |
| 36 | exp Aged/ |
| 37 | exp Geriatrics/ |
| 38 | (adult* or elderly* or geriatric*).mp. |
| 39 | 35 or 36 or 37 or 38 |
| 40 | limit 39 to "all adult (19 plus years)" |
| 41 | 39 or 40 |
| 42 | 34 and 41 |
| 43 | limit 42 to humans |
