# Supplementary material for: Effects of neuromuscular gait modification strategies on indicators of knee joint load in people with medial knee osteoarthritis: A systematic review and meta-analysis
Source: PLoS One. 2022 Sep 21;17(9):e0274874. doi: 10.1371/journal.pone.0274874 (PMC9491578; doi:10.1371/journal.pone.0274874)
Supplement: Attachment — Submitted filename: Response to reviewers.docx [file pone.0274874.s003.docx]

**Rebuttal in response to reviewers**

**Revision-** PONE-D-21-38506R1: Effects of neuromuscular gait modification strategies on indicators of knee joint load in people with medial knee osteoarthritis: A systematic review and meta-analysis

**Author’s name:** M Denika C Silva	**email:** [denika.silva@canberra.edu.au](mailto:denika.silva@canberra.edu.au)

We greatly appreciate your time in reviewing the manuscript and the detailed comments provided which are helpful to improve the paper.

Please see the following point-by-point responses to each comment raised by the academic editor.

Editor comments are in **black font** followed by the author's responses in **blue font.**

Please note the line numbers and page that we mention in this reply, refer to the manuscript with track changes.

**Journal Requirements:**

Please review your reference list to ensure that it is complete and correct. If you have cited papers that have been retracted, please include the rationale for doing so in the manuscript text, or remove these references and replace them with relevant current references. Any changes to the reference list should be mentioned in the rebuttal letter that accompanies your revised manuscript. If you need to cite a retracted article, indicate the article’s retracted status in the References list and also include a citation and full reference for the retraction notice.

**Author response:** We reviewed the reference list and can confirm that it is complete and correct. The reference list does not include any papers that have been retracted.

**Thank you very much to the editor, for the feedback and comments on the manuscript. The comments are very valuable and very much appreciated.**

**Editor Comments:**

"Statistical heterogeneity (I2) values were considered unreliable if less than seven papers were included in a meta-analysis [27].": My understanding is that it is not the I2 that is unreliable in this situation. The findings from your meta-analyses are unreliable if I2 is high. If you have small number of studies in a meta-analyses, then your I2 is likely to be biased (not unreliable). The terms "bias" and "reliable" reflect have different meanings.

**Author response:**

Thank you, yes the terms bias and reliability were being used too loosely. This has been revised now, in the methods “*Statistical heterogeneity (I^2^) values were estimated but may be biased if a smaller number of studies was included in meta-analyses [27]*.” (lines 170 to171)

In addition, this has been revised in the results. “*Statistical heterogeneity values of I^2^=0, may be biased as there were very few studies included (k=3) [27]*.” (lines 279 to 281)

**Editor comment:**
In my previous comments, I suggested you to discuss the clinical heterogeneity of included studies in meta-analyses. At that time, I wrote "You could have discussed the likely impact of heterogeneity between included studies from a clinical and methodological point of view." I still believe this is important and you could discuss this and how or whether clinical heterogeneity is likely to have impacted in your findings. If clinical heterogeneity may have impacted on findings, how should the reader interpret it. This paper discusses this and may be of interest: <https://bmcmedresmethodol.biomedcentral.com/track/pdf/10.1186/s12874-016-0121-7.pdf>.

**Author response:**

Thank you for the feedback and for recommending a useful paper on clinical heterogeneity.

In this review, we aimed to determine the effects of neuromuscular gait modification strategies on indicators of medial knee joint load in people with medial knee OA. Clinical variability characteristics include participants, the types or timing of outcome measurements, and the intervention (Cochrane handbook). All participants had medial knee OA, the severity of which, by study, is described in Table 3. We did not observe any effect due to KL grade, but the described ranges were wide. Other participant characteristics such as age and BMI varied, but not widely, since people with medial knee OA are a fairly homogenous population with older age and moderate to high BMI being a characteristic. Finally, although intervention timing was a primarily single session, there were longer-term interventions included. We did not analyse these separately because of the low number of studies. However, observation of the effect sizes did not reveal a marked differential effect. The intervention times are labelled on the forest plots and included in Table 4. Therefore, the reader can interpret the results of the meta-analyses in context.

The impact of clinical heterogeneity has now been included in our limitations (lines 457 to 470). *“**Moreover, the results of the meta-analyses of this review potentially have been affected by clinical heterogeneity of included studies; including treatment duration, dose, and whether specific KAM feedback is used. The main source of clinical heterogeneity is probably intervention time. However, the majority of studies reported single-session data, and the forest plots did not suggest that intervention time significantly altered the effect size. Therefore, the clinical implication is that a single session is likely to be effective, however, the longer-term effects of the strategies are not yet well understood. The effects of trunk lean and toe-out seem to be enhanced by larger angles and the effect of toe-in is likely enhanced by specific KAM feedback. Therefore, when applying the findings of this review to practice, clinical heterogeneity in intervention suggests that trunk lean and toe-out with greater angles, and toe-in with specific KAM feedback may be more effective.”*
